# Excess Cockerels in the Context of Legal Regulations, Animal Welfare and the Bioeconomy

**DOI:** 10.3390/ani16162459

**Published:** 2026-08-07

**Authors:** Hanna Spasowska, Umair Ahsan, Justyna Batkowska

**Affiliations:** 1 Department of Administrative Law and Administrative Science, Institute of Legal Sciences, Maria Curie-Sklodowska University (Lublin), 5 Maria Curie Sklodowska Sq., 20-031 Lublin, Poland; hanna.spasowska@mail.umcs.pl; 2 Department of Plant and Animal Production, School of Food, Agriculture and Livestock, Burdur Mehmet Akif Ersoy University, 15030 Burdur, Turkey; uahsan@mehmetakif.edu.tr; 3 Institute of Biological Basis of Animal Production, University of Life Sciences in Lublin, 13 Akademicka St., 20-950 Lublin, Poland

**Keywords:** animal welfare, bioeconomics, carbon dioxide culling, in ovo sexing, layer cockerels, legal framework

## Abstract

The global poultry industry faces a critical ethical and bioeconomic challenge regarding the annual culling of 6–7 billion day-old male layer chicks, which lack commercial value for mass meat production. This study critically evaluates European and global regulatory frameworks alongside the practical, economic, and ethical viability of alternative management strategies. While legislative bans in nations like Germany and France push for in ovo sexing technologies, and several systems are already operational across Europe, these methods currently impose substantial financial burdens and operational constraints that hinder their global commercial implementation. Alternative approaches, including rearing light-type cockerels or caponisation, frequently face significant sustainability challenges due to poor feed conversion ratios, prolonged production cycles, and inherent animal welfare concerns. Consequently, the immediate, worldwide integration of these technological alternatives remains challenging. This paper offers a pragmatic, bioeconomically stable recommendation: while in ovo systems continue to expand towards genuine high-throughput global viability, a sustainable approach—manual post-hatch sorting followed by immediate culling using methods permitted under current animal welfare legislation—may represent a transitional management option in jurisdictions where commercially viable in ovo sexing technologies are not yet available. Managed strictly as Category 3 animal by-products under Regulation (EC) No 1069/2009, the resulting material could potentially be directed to biogas plants to recover renewable energy. When implemented in accordance with Regulation (EC) No 1069/2009, such an approach may provide a legally compliant pathway for recovering renewable energy while remaining consistent with current veterinary and environmental regulations.

## Simple Summary

Every year, billions of male chicks from egg-laying chicken breeds are culled using methods permitted under current animal-welfare legislation immediately after hatching. Because they cannot lay eggs and grow too slowly for conventional meat markets, keeping them remains economically challenging under current mass-production models. This global practice raises significant ethical concerns. The aim of this study was to evaluate the current laws, social expectations, and proposed alternative solutions to this problem. Our analysis shows that while new technologies can determine the sex of a chick before it hatches and are already operating in some European countries, these methods still face financial and logistical challenges preventing their immediate worldwide adoption. Furthermore, raising these male chicks for food requires generally substantially greater feed inputs and longer production periods. We conclude that alongside the gradual expansion of egg-testing technologies, a legally compliant transitional approach for many regions might involve culling the hatched chicks using legally authorized methods. Where applicable, these by-products could potentially be utilized to generate renewable energy in biogas plants. This solution provides immediate value to society by balancing strict animal welfare and green energy goals with the survival of the farming sector, ensuring that eggs remain affordable for consumers without causing unnecessary suffering to the animals.

## 1. Introduction

The poultry industry generates significant quantities of organic by-products, requiring the implementation of new disposal technologies [1]. The earliest stage is chick hatching. Typically, the material produced here consists of unfertilised and unhatched eggs, eggshells, and culled diseased and deformed chicks (unsalable chicks). However, in commercial poultry, egg production is solely dependent on female layer birds that have been deliberately bred over decades for high frequency ovulation and long-term egg production. Male chicks from these breeds, primarily White Leghorn derivatives and brown commercial hybrids, are incapable of profitable meat production because their body conformation, daily weight gain, and feed conversion ratio differ significantly from standards of broiler breeds. Therefore, male layer chicks have no commercial value within the hatchery; they are classified as animal by-products upon culling and are usually culled within hours of hatching. The Food and Agriculture Organization of the United Nations, in its annual report, stated that the global laying hen population exceeds 7.6 billion birds and annual egg production surpasses 93 million tonnes [2]. Based on the sex ratio in commercial layer hatcheries, it is estimated that 6–7 billion male layer chicks are culled annually worldwide [3]. After chick sexing, (i.e., separating them by sex) [4], cockerels are culled mainly using mechanical devices or, less frequently, through the application of carbon dioxide. Such handling of chicks after hatching raises numerous ethical controversies. Nevertheless, it remains widely practiced in jurisdictions where commercially viable alternatives are not yet available due to the intensity of industrial layer farming, where the poultry house, often a cage system, is treated as an ‘egg factory’.

Beyond legal compliance and animal welfare considerations, the economic feasibility of alternative management strategies has become one of the principal determinants of their commercial implementation. Capital investment requirements, operating costs, hatchery throughput, feed efficiency and consumer acceptance increasingly influence decision-making within the poultry sector. Consequently, the present review evaluates available management strategies from an integrated legal, technological, biological and economic perspective.

The aim of this study was to critically evaluate the management of surplus layer-type cockerels by contrasting the current European and global regulatory frameworks with the economic, practical, and ethical viability of alternative technological solutions, ultimately proposing a pragmatic pathway aligned with both animal welfare and circular economy principles.

## 2. Literature Search and Selection Strategy

This manuscript constitutes a narrative review aimed at providing a critical synthesis of the literature, rather than a systematic review or meta-analysis. The scientific literature review was conducted using the Scopus and Web of Science databases, covering the period from 2000 to 2025. The following keyword strings and their combinations using AND/OR logical operators were applied: “day-old male chick culling” OR “layer cockerel management” AND “animal welfare”; “in ovo sexing” OR “in ovo sex determination” AND “poultry”; “caponisation” OR “capon” AND “layer cockerel”; “dual-purpose breeds” OR “dual purpose chicken” AND “egg production”; “Regulation (EC) No 1069/2009” OR “animal by-products” AND “poultry”; “CO_2_ euthanasia” OR “carbon dioxide culling” AND “chick”; as well as “bioeconomy” OR “circular economy” AND “poultry waste” OR “animal by-products”.

Additionally, backward citation tracking (snowballing) was employed, which involved screening the reference lists of key articles to identify further relevant publications. Inclusion criteria were strictly limited to peer-reviewed scientific articles published in English, fully available in open access, whose subject matter directly addressed layer-type cockerels, their culling, alternative management methods, or related legal and bioeconomic aspects. Excluded from the analysis were papers with only abstracts available, articles inaccessible under the open access model, publications in languages other than English, and monographs, book chapters, institutional reports, conference proceedings, and studies focusing solely on broiler chickens without reference to layer strains. An exception to the above criteria was made for historical sources, such as Masui and Hashimoto, published in 1933, which was cited due to their fundamental importance in describing traditional chick sexing methods, despite falling outside the adopted timeframe.

The analysis of legal acts was conducted based on the European Union’s legal information system, EUR-Lex, with particular emphasis on regulations, directives, and the case law of the Court of Justice of the European Union. Furthermore, Polish legal acts available in the Journal of Laws of the Republic of Poland were included. Regarding legal acts, no specific inclusion or exclusion criteria were applied, as the study utilised exclusively binding and applicable legal acts from the analysed period that have a direct impact on the management of surplus chicks. In terms of case law, judgments essential for the interpretation of the concept of ‘waste’ and the classification of animal by-products were cited, while the legal analysis also encompassed the impact of these regulations on the shaping of alternative technologies and market practices.

## 3. Regulatory Framework Governing Surplus Layer Cockerels

In public discourse and the available literature, the practice of killing day-old cockerels is frequently framed from the perspective of a waste problem [1,5,6,7,8,9]. However, an analysis of the current legislation leads to the conclusion that this view contradicts the established principles of environmental law. Polish and EU law indicate that such chicks, once killed, do not constitute waste sensu stricto, but are classified as animal by-products (ABPs), the management and disposal of which are subject to specific sanitary standards and animal welfare requirements [10,11]. Therefore, a critical analysis of this practice should be based on the interpretation of animal protection law, including welfare issues, rather than on arguments derived from waste law. This conclusion appears justified in light of Article 13 of the Treaty on the Functioning of the European Union, which recognises animals as sentient beings, necessitating the consideration of their welfare when formulating legislation and EU policies. Despite the economic justification for the practice, it is essential to respect the principles of humane treatment of animals, which implies seeking alternatives, such as innovative in ovo sexing technologies, allowing the identification of the embryos’ sex at an early stage of incubation and reducing the elimination of live chicks [12].

Accordingly, the legal classification of culled male chicks is not merely a terminological issue but determines the applicable regulatory regime, including welfare requirements, sanitary controls, and permissible recovery pathways. This distinction is particularly important when evaluating alternative management strategies within the framework of the circular economy, because different legal regimes impose different obligations regarding processing, transport, and final use of animal-derived material.

In European Union law, waste is defined in Directive 2008/98/EC of the European Parliament and of the Council, whose Article 3(1) declares that “‘waste’ means any substance or object which the holder discards or intends or is required to discard” [11]. Crucial to this definition is the adoption of the criterion of intent or the ‘obligation to discard’, as confirmed by the case law of the Court of Justice of the European Union (for instance, in cases C-129/96 Inter-Environnement Wallonie and C-9/00 Palin Granit) [13,14]. Directive 2008/98/EC does not apply to materials excluded under Article 2, including animal by-products, which are subject to specific regulation.

Within the European Union, animal by-products are regulated separately from waste under Regulation (EC) No 1069/2009, which establishes a harmonised framework governing their classification, collection, transport, processing and permitted end uses. This legal distinction is essential because it directly determines which management options are available for culled chicks.

According to the classification adopted in this legislative act (Article 10(a) and (k) of Regulation (EC)), Category 3 materials comprise raw materials considered to pose a relatively low risk to public health, yet still requiring controlled management. This category includes, inter alia, bodies or parts of animals killed for purposes other than human consumption and hatchery by-products, including day-old chicks culled for commercial reasons. Such classification determines that killed day-old chicks, including cockerels from the egg production sector, are assigned the lowest level of sanitary risk and are subject to a specific veterinary regime, without being designated as waste [10,15]. 

Nevertheless, Category 3 classification does not imply unrestricted management. Under Article 2(2)(a) of Directive 2008/98/EC and the corresponding provisions of Regulation (EC) No 1069/2009, animal by-products remain outside the scope of waste legislation only as long as they continue to be managed under the specific veterinary regime established by the ABP Regulation. Where Category 3 material is directed to biogas plants, composting facilities, incineration or landfill, additional legal requirements become applicable, creating an interface between veterinary legislation and environmental law rather than a complete substitution of one regime by the other.

Despite the adoption of such a low risk level, the EU legislator takes a precautionary approach, imposing an obligation to use strictly defined processing procedures (e.g., processing in approved plants, use in the production of feed for fur animals, or for energy production), which aims to limit potential epizootic threats and minimise environmental impact [16,17].

An essential element of the analysed legal regime is also the pursuit of maximising resource efficiency, which aligns with the concept of the circular economy. Indeed, EU regulations promote energy recovery and the secondary use of Category 3 materials, provided that stringent safety requirements are met, which allows for a reduction in waste volume and emissions associated with their disposal [18,19]. Consequently, the legal framework governing Category 3 material supports both sanitary protection and resource recovery. In practical terms, this enables the utilisation of culled chicks for renewable energy production in approved biogas facilities while maintaining compliance with veterinary safety standards.

Polish legislation follows the same regulatory approach by excluding animal by-products governed by Regulation (EC) No 1069/2009 from the general waste regime, except where specific waste-related provisions apply to recovery or disposal operations. Accordingly, the Polish legal framework does not differ materially from the European model in relation to the legal status of culled chicks.

**Global legislative approaches** outside the European Union vary significantly in both their regulatory scope and underlying axiological priorities. Within the United States legal framework, there is currently no comprehensive federal ban on the culling of day-old cockerels, reflecting a broader regulatory model that focuses primarily on food safety and production conditions, with a relatively limited scope for farm animal welfare protection. Crucially, the primary legislation in this area—the Animal Welfare Act—does not cover poultry, which is identified in legal doctrine as a significant systemic loophole [20,21]. Consequently, the regulation of this issue is largely fragmented and relies on non-legislative instruments, such as industry standards and private initiatives [22].

**The regulatory model in the United Kingdom** has developed differently; despite its departure from the European Union, the UK has maintained a high level of animal welfare protection, expanding its national regulations based on former EU standards. The Animal Welfare Act 2006 establishes general duties to ensure animal welfare, whereas specific regulations concerning poultry production and husbandry practices are detailed in secondary legislation and administrative guidelines. At the same time, the elimination of day-old cockerels remains a subject of regulatory debate, with current measures being largely gradual and predicated on close cooperation with the industry [23,24].

**Animal welfare frameworks in Japan** reflect the growing importance of this issue within public discourse and agricultural policy, although legal regulations there remain limited and evolutionary in nature. The Act on Welfare and Management of Animals, alongside initiatives by the Ministry of Agriculture, Forestry and Fisheries (MAFF), focuses primarily on promoting good husbandry practices and gradually raising standards, without introducing explicit prohibitions in the analysed area [25,26].

**The fragmentary regulatory direction in the People’s Republic of China** presents a particularly interesting case; despite the absence of a comprehensive animal welfare law, there is a discernible increase in the importance of this issue within the context of food safety and agricultural modernisation. Current regulations are scattered across various normative acts, with the primary emphasis placed on animal health and epizootic control rather than animal welfare sensu stricto [27,28].

**Comparative analysis reveals a clear dichotomy** between the European Union’s approach—aiming for the systemic elimination of practices deemed unethical—and the models adopted by certain third countries, where regulations are more heavily subordinated to production efficiency and food safety. Nevertheless, growing societal pressure and the evolution of international standards may lead to a gradual convergence of these systems towards higher animal welfare protection standards. Overall, outside the European Union, legal regulation generally focuses on animal health and food safety rather than prohibiting chick culling itself. Consequently, the principal regulatory pressure driving the development of in ovo sexing technologies currently originates from European welfare legislation. 

The regulatory developments discussed above also create new economic constraints for hatcheries. As legal restrictions on post-hatch culling expand, producers must increasingly evaluate alternative technologies not only according to their welfare benefits but also with respect to implementation costs, operational efficiency and long-term commercial viability.

## 4. Technological Solution

### 4.1. Traditional Procedures for One-Day-Old Laying Cocks

As previously mentioned, day-old layer cockerels, which effectively constitute a by-product from the production of commercial layer flocks, are, in the vast majority of cases, subject to disposal. Under EU Council Regulation (EC) No 1099/2009, approved culling methods include mechanical maceration at blade speeds ≥300 rpm and high-concentration CO_2_ gassing, which are methods permitted under the applicable legislation [29]. 

Ethical concerns are being increasingly raised about killing billions of healthy chicks for financial profitability. Germany became the first country to pass a legal prohibition on routine male layer chicks culling through an amendment to the Tierschutzgesetz [30]. While initial legislative plans targeted a strict day-7 limit, current regulations prohibit the termination of male embryos from incubation day 13 onward. This threshold aligns with updated neurobiological evidence, which indicates that the prerequisite pain-processing thalamocortical circuits are not yet functional before this stage [31,32]. France also adopted a similar prohibition, effective January 2023. Austria, Belgium, and Italy are in different stages of legislative development, and the European Commission’s Farm to Fork Strategy [33] lists male chick culling as a priority welfare concern.

It must be borne in mind, however, that new technologies, regardless of their utility, simultaneously impose a significant economic burden, both due to their cost and the extension or expansion of hatchery procedures. The commercial effects of this legislative change are significant. Large European layer hatcheries that produce 500,000 to 2,000,000 eggs per week must now either invest in validated ovo sexing technology or exit the industry for regulatory compliant egg production. This has resulted in an unparalleled convergence of poultry science, molecular biology, optical physics, and machine learning for the development of practical, high-throughput sex determination systems. Simultaneously, in the majority of worldwide markets where such regulatory pressure does not exist, manual post-hatch methods continue to be the dominant, cost-effective, and operationally proven standard.

### 4.2. Manual Post-Hatch Sexing Methods

Manual chick sexing methods such as vent sexing, feather sexing, and color autosexing are performed on day old chicks and rely on morphological or pigmentation traits that are phenotypically displayed at the time of hatching. These approaches do not require any laboratory equipment, molecular reagents, or complex instruments, making them operationally simple, readily scalable, and extremely cost-effective.

**Vent sexing** (cloacal examination) is the oldest commercial chick sexing technique, with its descriptive anatomical foundation still guiding professional training today. Sex is determined at the proctodeum level by visually or tactilely evaluating the cloacal eminence, which represents the only morphological difference between male and female chicks. The vent sexing procedure consists of holding the chick under intense lighting, applying gentle abdominal pressure to clear intestinal contents, and everting the proctodeum to evaluate the cloacal eminence [34,35,36]. Among all the sexing methods, the cost per chick is the lowest because of a higher dependency on labour rather than on capital equipment. However, achieving expert competency requires years of hatchery experience, with professional qualifications demanding months of supervised practice. Despite this, vent sexing remains the main technique used in commercial layer hatcheries throughout major global markets [37]. 

Feather sexing is based on a sex-linked difference in the rate of primary wing feather development, enabling rapid visual sex determination at hatch without cloacal examination [38]. Compared to vent sexing, feather sexing is faster and easier to implement routinely in commercial hatcheries, as it requires minimal training and avoids cloacal eversion [39,40]. However, its reliable performance depends heavily on maintaining the strict genetic purity of the breeder line and molecular verification of feathering genotypes [38]. Although feather sexing occurs post-hatch and is thus incompatible with legislation in countries like Germany and France, it remains in use for specific systems across markets lacking such regulatory pressure during the transition towards in ovo technologies [34]. 

**Colour autosexing** utilizes sex-linked plumage pigmentation genes to produce male and female chicks with distinguishable down colour at hatch, enabling immediate visual sex determination [41]. In commercial autosexing crosses, this allows for rapid performance with minimal operator training. While this method achieves high accuracy and reduces handling stress compared to vent sexing, reliable performance depends on strictly maintaining breeding line genetics to prevent unwanted variation [40]. Because colour autosexing is inherently a post-hatch method, it faces the same regulatory limitations as feather and vent sexing in areas adopting legislation that promotes pre-hatch sex determination and the reduction in male chick culling through in ovo techniques [42,43].

## 5. Alternative Technological Solutions

### 5.1. Molecular and Biochemical Methods

Molecular and biochemical methods of sex determination leverage the detection of sex-specific genetic, hormonal, and spectroscopic signals, offering a more accurate and reliable alternative to traditional phenotypic approaches, particularly in sexually monomorphic species.

**PCR-based genetic sexing** is founded on the avian sex chromosomes, which are ZZ for males and ZW for females. Molecular approaches utilise chromosome-specific markers, considering the differences between the homogametic sex (males) and heterogametic sex (females) [44]. It functions through conserved CHD (chromo-helicase-DNA-binding) genes found on avian’ sex chromosomes. Because the CHD-W gene is found on the W chromosome, only females have it. The other gene, CHD-Z, is present in both sexes (male, ZZ; female, ZW) because it is located on the Z chromosome. PCR using a single set of primers is used in the test. It amplifies homologous sections of both genes and incorporates introns whose lengths usually differ. Using P2/P8 or similar primers, PCR amplification of CHD1 genes produces two bands for females (ZW) and one band for males (ZZ). Males have a single CHD-Z band on a gel, whereas females have a second, unique CHD-W band [45]. More recent primers for female detection focus on W-specific genes such as SWIM or Xho-I. In the laboratory settings, PCR sexing on a drop of blood, feather pulp, or even embryonic tissue can produce results with approximately 100% accuracy. It is highly dependable and works on any breed of chicken. When sexual dimorphism is not visible at birth, molecular sexing of bird species is an important tool for conservation, breeding, and selection. This technique allows males to be distinguished from females at a very young age without causing the animals any stress [46,47].

**LAMP and isothermal assays**, such as RPA (recombinase polymerase amplification) and loop-mediated isothermal amplification (LAMP), were developed to simplify molecular sexing as rapid, simple, and economical diagnostic tools. There are various protocols for sexing using LAMP techniques, but the most reliable is identification based on Loop-mediated isothermal amplification (LAMP)-clustered regularly interspaced short palindromic repeats (CRISPR)/CRISPR-associated protein 12a (Cas12a). Using specific primers and sgRNA, it targets the W chromosome-specific segment EE0.6 and the chicken Z chromosomal gene DMRT1. Cas12a cleaves the LAMP amplicon to produce a fluorescent product that can be detected by a portable light device. This provides significant potential for determining the sex of chicken embryos at a very early stage of development [48]. These assays are potentially portable, faster, and easier than PCR (only a heater is required). However, egg samples, reagents, and certain equipment are still needed for accurate findings. Although it is not yet standard practice for sorting chicks in large quantities, it shows promise for in ovo testing where slower techniques are acceptable [47].

**Biochemical (hormone) assays** utilise sex-specific hormones for identification purposes. Sex steroids, which are secreted into the blood, are metabolised in the liver and, after being eliminated by the kidneys, build up in the embryonic urine or allantoic fluid [49]. Female-specific hormones, such as estrone sulphate, can be detected in egg fluid using an allantoic fluid assay. On day nine of incubation, commercial devices (such as Seleggt/Respeggt in Germany) collect egg fluid and perform an ELISA or DNA-based test for a female marker. Males send out a negative signal, while females send out a positive one, frequently with a hue shift. Such biochemical sexing can be more than 95% accurate. Respeggt, for instance, claims 99.5% accuracy. Although this technique is extremely precise and can be completed prior to hatching, preventing the birth of male chicks, specialised test kits are expensive per egg, and piercing the egg and extracting fluid requires skill, while handling may marginally lower the hatch rate [47].

### 5.2. Advanced Sexing Techniques

The implementation of advanced sexing techniques must be carefully evaluated based on the specific incubation day at which sex determination is performed. To clarify the biological and regulatory significance of these time points, day 7 is associated with the multisynaptic reflex arc closure, representing a highly precautionary threshold adopted by some legislative models [50]. However, days 12 to 13 mark the onset of measurable encephalogram (EEG) signals, which are scientifically considered the prerequisite for conscious pain perception. Therefore, in ovo sorting after day 13 raises significant welfare and regulatory acceptance concerns, which is consistent with current evidence on the onset of embryonic pain perception [50]. This consensus is also reflected in the legal regulations governing the use of animals for scientific and educational purposes, under which manipulations on avian embryos up to the last third of their normal development (i.e., prior to day 14 of a standard 21-day incubation) are not classified as procedures that compromise animal welfare [51]. However, as embryonic pain perception remains an area of active scientific discussion, it is important to emphasize that these developmental thresholds are based on currently available evidence and precautionary regulatory approaches, rather than on universally accepted biological cut-off points. Acknowledging this remaining scientific uncertainty is essential when establishing definitive legislative boundaries.

**Fourier transform infrared spectroscopy** (FT-IR) is a suitable tool for a quick, objective determination of a bird’s sex. The technique relies on variations in chromosomal size. Female birds have one W chromosome and one Z chromosome, while male birds have two Z chromosomes. The W chromosome has approximately 26,000,000 bp, while each Z chromosome has approximately 75,000,000 bp. However, rather than directly identifying this discrepancy in chromosomal size, FT-IR spectroscopy detects the molecular and spectroscopic differences associated with these sex-related biological characteristics, such as variations in total DNA content. Spectra can be recorded from germ cells obtained from the feather pulp of chicks as well as from the germinal disk of fertilized but unincubated eggs (day 0). Male and female bird cells differ significantly in the area of phosphate vibrations between 1080 and 1120 cm^−1^ [52,53]. Fourier transform infrared (FT-IR) spectroscopy converts raw data (interferogram) into actual spectra using a mathematical procedure called the Fourier transform. Infrared transmission and absorption spectra can provide molecular information on avian sex from pulp cells extracted from the feather shaft.

**Raman spectroscopy** allows the non-invasive and contactless determination of the sex of domestic chicken embryos in ovo as early as day 3.5 of incubation. This technique analyses the spectra of blood circulating in the extra-embryonic vessels and achieves a sexing accuracy of approximately 90%. Because the measurement is damage-free, hatchability is rarely affected. Importantly, sex determination is performed before the onset of embryonic sensitivity, making this approach a promising alternative to the culling of day-old male chicks in the laying hen industry [54]. Sex differentiation is possible due to the distinct Raman fingerprints of male and female embryos, which reflect sex-specific molecular characteristics. Variations in molecular vibrations, particularly DNA- and protein-related Raman bands, are analysed and classified as male or female using a support vector machine (SVM), a supervised machine learning classification method [55].

**Hyperspectral imaging** is utilised by commercial companies, such as Agri Advanced Technologies (AAT, Germany), to scan eggs on day 13 of incubation. Male and female chicks of certain layer breeds have differently coloured down feathers (e.g., white vs. brown) when candled; hyperspectral sensors detect this and yield approximately 98–99% accuracy. According to reports, the accuracy of AAT’s technique on brown-layer eggs is approximately 98.8%. Using multispectral reflectance, another project (Project Soo in Tronico, France) reported an accuracy of approximately 90% by day 9 of incubation. At 30,000–50,000 eggs per hour, Hypereye (Canada) is aiming for 99% accuracy. Once set up, these contactless optical devices can process eggs quickly without damaging them. However, they primarily only function for breeds that have sex-linked plumage differences, which are widespread in layers but not in broilers [47].

**3D X-ray microcomputed tomography** (Micro-CT) is a three-dimensional imaging technique that employs X-rays to visualise an object’s internal structure slice by slice. Unincubated eggs (day 0) can be sexed in ovo by molecular genetic analysis of dermal blastocysts; however, for precisely directed cell biopsies, it is essential to identify the exact position of the germinal disc. In theory, microcomputed tomography can be used to locate the germinal disc in unincubated eggs without causing any harm to the eggshell. There are no known detrimental effects on the hatchability of irradiated eggs or on embryonic development [43,56].

**Magnetic resonance imaging** (MRI) enables the clear visualisation of embryonic structures by day 12, serving as the basis for a non-invasive technique for in ovo sexing of domestic chicken eggs [57]. MRI sequences, especially T1-weighted images, can clearly identify the albumen, yolk sac, allantoic sac, and amniotic cavity in addition to the embryo. Driven by advancements in artificial intelligence, this technology has progressed significantly beyond the initial research stage. Automated MRI-based platforms coupled with AI image recognition (such as the Genus Focus system by Orbem) are currently operational at several large commercial hatcheries in Europe. Although initial capital costs and processing speeds remain important logistical considerations when comparing these systems to traditional methods [43,47], AI-assisted MRI has demonstrated successful commercial integration and high-throughput capabilities.

## 6. Economic Feasibility of Alternative Management Strategies for Surplus Layer-Type Cockerels

Although rearing layer-type cockerels eliminates the ethical concerns associated with chick culling, its practical implementation remains strongly dependent on production economics. The traditional sexing methods described above necessitate the disposal (culling) of either the cockerels themselves (light-type males) or the eggs containing developing male embryos. However, public opinion considers the rearing of these birds more humane, particularly in less intensive systems [58] that permit the presence of roosters within layer flocks. In this context, cockerels can be utilised in two ways: first, as breeding stock for producing chicks on-farm, and secondly, for meat production, primarily for self-supply. Such rearing requires an extended period as well as less intensive nutrition, owing to a slower growth rate compared to typical meat-type birds. A further aspect is the colourful plumage of these birds; keeping them on organic and agrotourism farms can significantly enhance the attractiveness of the enterprise [59]. However, the question arises as to whether the economic benefits of these birds, as well as the quantity and quality of the meat obtained from them, will be sufficient to justify keeping them. 

### 6.1. Economic Implications of Conventional Post-Hatch Chick Culling

The economic evaluation of alternatives to post-hatch chick culling should be considered within the broader context of the expanding global egg industry. According to the OECD–FAO Agricultural Outlook 2022–2031, global egg production is expected to continue increasing in response to population growth, urbanisation and rising demand for affordable animal protein. Simultaneously, poultry producers face increasing pressure associated with feed prices, energy costs and market volatility, making resource efficiency and production optimisation central determinants of competitiveness [60]. 

Historically, the culling of newly hatched male layer chicks represented the economically preferred solution because specialised layer strains have been intensively selected for egg production rather than meat yield. Consequently, male chicks exhibit poor growth performance, inefficient feed conversion and low carcass value, rendering their commercial rearing economically unattractive under conventional production systems [7].

Although post-hatch culling requires relatively low direct operating costs, its apparent economic efficiency is limited to hatchery-level expenditure. Approximately half of all incubated eggs develop into male embryos, which consume identical incubation resources—including energy, labour, equipment capacity and technical supervision—before being removed immediately after hatching. Consequently, substantial biological and technical resources are invested without generating corresponding economic returns. From a production economics perspective, this represents inefficient allocation of hatchery resources rather than optimal resource utilisation.

Moreover, increasing legislative restrictions on chick culling fundamentally alter the economic rationale underlying this traditional practice. Compliance with emerging animal welfare legislation requires hatcheries to evaluate management strategies according to long-term regulatory certainty, technological adaptability and commercial resilience rather than immediate operational costs alone. Therefore, the economic attractiveness of conventional chick culling is progressively declining despite its historical dominance.

### 6.2. Economic Performance of In Ovo Sexing Technologies

Among currently available alternatives, in ovo sex determination has emerged as the most technologically advanced strategy for replacing conventional chick culling. By identifying embryo sex before hatching, these technologies reduce unnecessary investment in economically non-productive embryos while maintaining the productivity of specialised laying strains [42].

Commercial implementation, however, requires considerable capital investment. Automated sampling systems, analytical equipment, software integration and quality-control procedures increase both capital expenditure (CAPEX) and operating expenditure (OPEX) compared with conventional hatchery management. Consequently, economic feasibility depends not only on diagnostic accuracy but also on implementation costs, analytical throughput, operational reliability and compatibility with existing hatchery infrastructure [42].

Current technologies differ substantially regarding analytical principles, timing of embryo sex determination, automation potential and commercial readiness. Earlier diagnosis enables hatcheries to reduce unnecessary incubation costs by limiting the consumption of electricity, incubator capacity and labour associated with male embryos. Conversely, later diagnosis reduces these economic benefits because non-productive embryos continue to occupy production capacity for longer periods [42].

Successful commercial implementation additionally depends on processing capacity. Industrial hatcheries process hundreds of thousands of eggs per production cycle; therefore, diagnostic systems must combine high analytical accuracy with throughput compatible with large-scale commercial operations. Accordingly, technological maturity should be evaluated not only according to laboratory performance but also according to industrial scalability and operational efficiency [42].

Beyond implementation costs, in ovo technologies should also be evaluated from the perspective of resource efficiency throughout the production chain. Conventional incubation of embryos subsequently culled after hatching requires continuous consumption of energy, labour and incubator capacity without generating commercial value. Life-cycle assessment studies demonstrate that poultry production efficiency depends upon cumulative resource use, including energy consumption, infrastructure utilisation, transport and waste management [8]. Consequently, reducing unnecessary biological losses during incubation may improve both economic efficiency and overall sustainability. Rather than focusing exclusively on immediate implementation costs, economic assessments should therefore incorporate resource efficiency across the entire production system [8].

Although current technologies continue to evolve, available evidence indicates that in ovo sex determination offers the greatest long-term potential for reconciling economic performance with animal welfare requirements. Continued improvements in automation, analytical throughput and implementation costs are expected to enhance commercial competitiveness as adoption increases [42].

### 6.3. Economic Aspects of Rearing Layer-Type Cockerels

Rearing layer-type male chicks represents the most direct ethical alternative to post-hatch chick culling because it completely eliminates the need for euthanasia of healthy animals. Nevertheless, its commercial viability remains constrained by biological performance. Compared with specialised broilers, layer cockerels exhibit slower growth, poorer feed conversion efficiency, lower carcass yield and prolonged production cycles, resulting in substantially higher production costs per kilogram of meat [7].

Feed constitutes the largest component of poultry production costs, accounting for approximately 60–70% of total production expenditure in intensive poultry systems (OECD & FAO, 2022) [60]. Consequently, inferior feed efficiency significantly reduces the economic competitiveness of layer cockerel production. Longer fattening periods additionally increase housing, labour and energy costs while reducing annual housing turnover and production capacity.

Commercial viability therefore depends largely upon market differentiation rather than biological productivity. Premium market segments, welfare-oriented certification schemes and consumer willingness to pay higher prices may partially compensate producers for increased production costs. Without such price premiums, however, the production of layer cockerels remains economically disadvantaged compared with conventional broiler production [61].

### 6.4. Market Acceptance and Commercial Implementation

Consumer acceptance constitutes an increasingly important determinant of economic feasibility. Surveys consistently demonstrate that consumers generally oppose the routine culling of day-old male chicks once informed about current hatchery practices and express greater acceptance of alternative production systems [61].

Nevertheless, willingness to pay (WTP) remains conditional rather than unlimited. Although many consumers declare readiness to pay moderate price premiums for eggs produced without chick culling, purchasing decisions continue to depend on retail price, product quality, confidence in certification systems and transparency of production methods. Consequently, positive consumer attitudes do not necessarily translate into proportional market demand, creating uncertainty regarding the commercial implementation of welfare-oriented production systems [61].

Retailers further influence market development through voluntary animal welfare standards and private certification schemes. In several European countries, retail chains have committed themselves to sourcing eggs from production systems eliminating post-hatch chick culling, thereby accelerating technological innovation independently of statutory legal requirements. These initiatives reduce investment uncertainty while strengthening commercial incentives for the adoption of alternative technologies.

### 6.5. Integrating Legal Requirements with Economic Feasibility

The transition away from conventional chick culling demonstrates the increasing interdependence of legal regulation, technological innovation and production economics. Legislative restrictions alter the economic environment by reducing the feasibility of traditional management practices while simultaneously encouraging investment in alternative technologies.

Economic feasibility should therefore be evaluated through an integrated framework encompassing legal compliance, technological maturity, biological efficiency and market acceptance. No currently available strategy fully satisfies all criteria simultaneously. Conventional chick culling remains operationally simple but faces increasing legal restrictions. Rearing layer cockerels substantially improves animal welfare but entails higher production costs. In contrast, in ovo sex determination combines legal compatibility with preservation of specialised layer genetics, although implementation costs remain an important barrier to widespread adoption [42].

Overall, future competitiveness of the egg sector will depend on the successful integration of regulatory compliance, economic sustainability, technological innovation and societal expectations. Consequently, economic feasibility should not be regarded solely as a question of production costs but as a multidimensional concept encompassing resource efficiency, long-term investment security, consumer acceptance and regulatory resilience.

To provide a comprehensive overview of the available alternatives, Table 1 summarises the key economic, technological, biological, and welfare dimensions of the various management strategies for surplus layer-type cockerels, based on a synthesis of the current scientific literature.

## 7. Other Possibilities

**Caponisation** as a management strategy for surplus layer-type cockerels and local poultry breeds has frequently been highlighted as a traditional utilisation method [65,66]. The procedure involves the surgical removal of the testes from young cockerels, typically before they reach sexual maturity, to enhance carcass traits (such as carcass weight), increase intramuscular fat deposition, and improve overall meat quality attributes, namely juiciness and tenderness. Consequently, capon meat is highly regarded for its superior sensory characteristics. The underlying mechanism driving these alterations in meat quality is the disruption of circulating androgen levels following gonadectomy, as these hormones play a pivotal role in avian lipid metabolism [67]. Moreover, the economic value of capon production is largely restricted to regional premium markets and therefore cannot be regarded as a scalable alternative for commercial egg production.

Animal welfare concerns and legislative frameworks surrounding caponisation have drawn increasing scrutiny, despite the practice being an ancient tradition with deep breeding, cultural, and culinary roots across many European Union member states, including Poland [68,69,70]. Historically, and currently in various regions, the surgical procedure is performed without anaesthesia while the bird is manually restrained or secured to a table [71]. This practice unequivocally compromises physical welfare, inducing acute pain, discomfort, and a high risk of postoperative infection, which has led to widespread criticism in both public and scientific domains as being incompatible with modern welfare standards. Consequently, EU member states have diverged into two distinct regulatory stances: a total prohibition of the practice or conditional allowance under strict criteria, aligned with broader principles such as those outlined in Directive 2010/63/EU regarding animal protection [72].

National regulatory approaches to caponisation vary significantly across Europe. Under the Austrian Animal Protection Act (Tierschutzgesetz [30]), surgical interventions on animals are strictly prohibited unless medically indicated or essential for specific breeding objectives, rendering caponisation illegal due to the lack of direct medical necessity. An identical approach is enforced in the Netherlands, where the Animal Welfare Act (Wet dieren [73]) bans the infliction of pain or suffering without a justified cause. Conversely, countries like Italy, Poland, and France permit the practice under restrictive conditions. In Italy, while the Penal Code (Codice Penale [74]) penalises animal cruelty, caponisation is tolerated provided it is executed to minimise pain and suffering. Similarly, the Polish Animal Protection Act [75] permits the procedure but mandates that it be performed by appropriately qualified personnel making every effort to mitigate pain and stress. In France, the castration of roosters persists as a prized element of traditional poultry husbandry in regions such as Bresse, Gers, and Vendée, where it delivers premium products like the chapon de Bresse, which holds an Appellation d’Origine Contrôlée (AOC) status; nevertheless, French regulations strictly stipulate that the procedure must only be carried out by qualified or specifically trained operators.

## 8. Socio-Political Drivers and Legislative Models of Chick Culling Regulations

**The economic dilemma** in poultry breeding faced by many countries stems from the fact that layer-type cockerels hold no market value for egg production or mass rearing, leading to their culling immediately post-hatch. Beyond the regulatory framework, it is justified to anchor these solutions within the broader context of sustainable development and the shaping of ethical consumption patterns. The contemporary approach to agri-food sector regulation clearly shifts from a purely productionist model to a holistic perspective that integrates environmental, social, and economic goals, as reflected in both European Union strategic documents and academic doctrine [76,77]. From this viewpoint, animal welfare represents not only a subject of statutory regulation but also an essential component of socially responsible production and consumption.

**Public acceptance of agricultural technologies** and practices undeniably acts as a determining factor for both the pace of their implementation and the development of the market for animal-derived products. As indicated in the literature, rising consumer awareness regarding animal welfare translates into increased demand for products that meet higher ethical standards, which in turn exerts regulatory pressure on legislators and market participants [78,79]. This mechanism functions as a feedback loop: societal norms influence legislation, while legislation institutionalises specific ethical standards, reinforcing their internalisation within economic practice.

The systematic tightening of legal regulations aimed at eliminating practices deemed unethical, such as the mass culling of day-old cockerels, is a direct consequence of these shifting dynamics. In its scientific opinions, the European Food Safety Authority (EFSA) emphasises that procedures causing animal suffering that are not justified by overriding reasons should be phased out, and that replacing them with alternative technological solutions is a vital element of elevating welfare standards [12,80]. In this context, the development of technologies such as in ovo embryo sexing aligns not only with the innovation framework of the agricultural sector but also with the fulfilment of public expectations regarding the humane treatment of animals. From a systemic perspective, the effectiveness of legal regulations in the field of animal welfare is strictly correlated with the level of social acceptance and the degree to which ethical values are internalised by consumers. Integrating legal instruments with educational, informational, and market-driven initiatives (such as welfare certification schemes) can facilitate a permanent paradigm shift in production and consumption towards a more sustainable and responsible direction.

The German and French legislative reforms illustrate how welfare policy increasingly functions as an innovation driver. Rather than merely prohibiting chick culling, both regulatory systems create strong economic incentives for the commercial deployment of in ovo sexing technologies. Consequently, legal intervention affects not only animal welfare standards but also investment decisions, technological development and the future competitiveness of the poultry sector.

## 9. Conclusions

From a regulatory standpoint, the legal classification of culled male chicks as Category 3 animal by-products under Regulation (EC) No 1069/2009, rather than as waste within the meaning of Directive 2008/98/EC, establishes the foundational legal framework governing permissible management pathways (Regulation (EC) No 1069/2009, Art. 3(1), Art. 10; Directive 2008/98/EC, Art. 2(2)(b)). This regulatory distinction, widely recognised in both policy and the academic literature [33,81], has direct practical implications for determining applicable sanitary requirements, processing obligations, and recovery options, including directed use in biogas facilities. The legislative reforms enacted in Germany and France demonstrate that welfare-driven legal intervention can function simultaneously as an innovation driver, accelerating investment in alternative technologies and reshaping the economic environment of the egg sector.

From a technological perspective, in ovo sex determination has emerged as the most promising commercially scalable alternative to conventional post-hatch culling. Several systems are currently operational in commercial hatcheries across Europe, offering sexing accuracies exceeding 95% at throughput rates compatible with industrial-scale production. Nevertheless, implementation costs, breed-specific applicability, and the developmental stage at which sexing is performed remain important determinants of commercial viability. Technologies performing sex determination prior to the potential onset of embryonic pain perception are preferable from both a welfare and a regulatory acceptance perspective. It is important to clarify that day 7 represents a highly precautionary threshold associated with early neural development, whereas days 12 to 13 relate to later neurophysiological development potentially relevant to conscious perception. These time points should not be presented as universally established biological thresholds.

From an economic perspective, the viability of alternatives to chick culling is increasingly supported by evolving consumer preferences. Multiple studies conducted across European markets consistently demonstrate consumer willingness to pay price premiums for products certified as produced without post-hatch chick culling, suggesting that market mechanisms may partially offset implementation costs under appropriate labelling and communication frameworks. Simultaneously, resource efficiency analyses indicate that elimination of unnecessary incubation of non-productive male embryos may generate operational savings that partially mitigate capital expenditure associated with in ovo sexing systems.

Rearing layer-type cockerels constitutes a welfare-positive alternative, but its commercial scalability remains constrained by poor feed conversion efficiency, extended fattening periods and limited market differentiation. Its practical applicability is therefore confined to premium and niche market segments rather than mass commercial production. Caponisation, while carrying historical and cultural significance in specific regional contexts, cannot be regarded as a commercially scalable strategy given the associated animal welfare concerns and legislative restrictions increasingly applicable in European jurisdictions.

Overall, the evidence reviewed does not support a single universally optimal management strategy. Rather, the most appropriate pathway depends on hatchery scale, breed characteristics, available technology, regulatory jurisdiction and market positioning. Where in ovo sexing technologies compatible with early-stage embryo determination are commercially accessible, their adoption is strongly supported by the convergence of welfare legislation, consumer demand and long-term economic considerations. Under specific circumstances, and depending on local regulatory and economic conditions, in jurisdictions or production contexts where such technologies have not yet achieved operational viability, post-hatch sorting followed by immediate culling using methods considered acceptable under current animal welfare standards, including high-concentration carbon dioxide, could provide a legally compliant transitional approach. This may represent a viable option, provided that the resulting Category 3 material is directed to approved recovery pathways, including biogas production, in accordance with Regulation (EC) No 1069/2009.

Further regulatory harmonisation at the European Union level, aligned with the objectives of the Farm to Fork strategy, is expected to accelerate the commercial deployment of in ovo sexing technologies while creating consistent market conditions across Member States. Continued interdisciplinary research integrating legal analysis, technological development, biological performance assessment and economic modelling will be essential to support evidence-based policy development in this rapidly evolving field.

## Figures and Tables

**Table 1:** Comparative assessment of management strategies for surplus layer-type cockerels: economic, technological and welfare dimensions.

Parameter	Post-Hatch Culling (CO_2_)	In Ovo Sexing (Early; ≤d4)	In Ovo Sexing (Late; d9–d13)	Rearing Layer Cockerels	Dual-Purpose Breeds	Caponisation
A. Biological performance ***						
Growth rate (g/day)	n/a	n/a	n/a	9–20 [62]	25–40 [63]	~20–30 [64]
Feed conversion ratio (FCR)	n/a	n/a	n/a	4.0–10.0 [62]	3.0–4.5 [63]	4.0–6.0 [64]
Fattening period (weeks)	n/a	n/a	n/a	8.5–18 [62]	12–16 [63]	20–30 [64]
Final body weight (kg)	n/a	n/a	n/a	1.3–1.5 [62]	2.5–3.5 [63]	2.5–3.5 [64]
B. Technological characteristics						
Sexing accuracy (%)	>99 [42]	95–99 [42]	95–99 [42]	n/a	n/a	n/a
Throughput (eggs or chicks/h)	High	3000–20,000 [42]	3000–20,000 [42]	n/a	n/a	n/a
Commercial availability (EU)	Yes	Yes (5 systems) [42]	Yes (5 systems) [42]	Limited [62]	Limited [63]	Very limited [64]
Pain perception risk (embryo)	n/a	None (<d7) [42] *	Possible (≥d7–d12) [42] *	n/a	n/a	Yes (surgical) [64]
C. Economic indicators						
Direct implementation cost	Low	High (CAPEX) [42]	High (CAPEX) [42]	Moderate	High (breeding) [63]	Moderate
Incubation resource efficiency	~50% loss [42]	Optimised	Partial	n/a	n/a	n/a
Feed cost as share of production cost (%)	n/a	n/a	n/a	60–70 [60]	60–70 [60]	60–70 [60]
Consumer WTP premium (eggs; relative)	None	+100% [37]	+100% [37]	+29% (CH) [61] **	+29% (CH) [61] **	n/a
Consumer WTP premium (meat; absolute)	None	n/a	n/a	+€1–2/2 burgers [37]	+13% (CH) [61]	n/a
Scalability for mass commercial production	High	High [42]	High [42]	Low [62]	Moderate [63]	Very low
D. Legal and welfare status (EU)						
Current legal status (EU)	Permitted; restricted DE/FR	Compliant	Compliant	Compliant	Compliant	Restricted (Dir. 2010/63/EU)
ABP classification (Reg. EC 1069/2009)	Category 3	Category 3 (male eggs)	Category 3 (male eggs)	n/a	n/a	n/a
Animal welfare assessment	Acceptable (current standards)	High (pre-pain)	Moderate (pain risk)	High	High	Low (surgical stress)

Favourable/Moderate/Unfavourable; n/a = not applicable; CH = Switzerland; DE = Germany; FR = France; ABP = animal by-product; CAPEX = capital expenditure; WTP = willingness to pay; FCR = feed conversion ratio; * Estimated onset of pain perception ≥d7 (multisynaptic reflex arc closure) to d12–d13 (encephalogram signal); ** WTP for dual-purpose eggs: CHF 4.39 vs. CHF 3.40 (conventional); WTP for DP chicken breast: CHF 37.4 vs. CHF 33/kg; *** Data presented in Section A represent synthesized ranges derived from the cited literature to provide a generalized comparative overview rather than specific trial results.

## Data Availability

The data presented in this study are available on request from the corresponding author.
